# Effects on annual income changes after radical radiotherapy versus after prostatectomy in patients with localized prostate cancer with a specific employment status: A web-based pilot study

**DOI:** 10.1371/journal.pone.0258116

**Published:** 2021-09-30

**Authors:** Masanari Minamitani, Tomoya Mukai, Hideomi Yamashita, Atsuto Katano, Keiichi Nakagawa

**Affiliations:** 1 Department of Radiology, The University of Tokyo Hospital, Tokyo, Japan; 2 Graduate Schools for Law and Politics, The University of Tokyo, Tokyo, Japan; 3 Department of Comprehensive Radiation Oncology, Graduate School of Medicine, The University of Tokyo, Tokyo, Japan; University of Nebraska Medical Center, UNITED STATES

## Abstract

Men with localized prostate cancers are insured for undergoing radical radiotherapy or prostatectomy. However, limited information is available on the influence of cancer treatments on patients’ employment status in Japan. Therefore, in this web-based survey, we aimed to compare the effects of post-treatment changes on the annual income of patients with prostate cancer after undergoing radical radiotherapy and prostatectomy and to identify the risk factors associated with the decrease in annual income. We investigated the clinical characteristics and demographics including pre-treatment working status, self-employment, non-regular employment, working for wage or salary, and joblessness of patients with localized prostate cancer. Multivariable logistic regression was performed to analyze the effects of various factors on the change in the annual income of self-employed and non-regularly employed workers. Seventy-eight eligible patients with localized prostate cancer had undergone radiotherapy, and 128 patients had undergone prostatectomy. Among self-employed and non-regularly employed workers, post-treatment income decline rates in those who underwent radiotherapy were smaller but not significant (12% vs. 42%, P = 0.074). Multivariable logistic regression analysis revealed that initial treatment for prostate cancer was the only significant risk factor for the post-treatment income decline among self-employed and non-regularly employed workers. Radiotherapy was associated with a smaller decrease in income (odds ratio, 0.22; 95% confidence interval, 0.052–0.95; P = 0.042). Our novel results implied the effectiveness of radiotherapy in preventing post-treatment income decline among patients with prostate cancer based on specific employment status: self-employed or non-regularly employed.

## Introduction

Prostate cancer is the most common cancer in men; in Japan, approximately 90,000 new cases were identified in 2017 [1]. Men with localized prostate cancer are often suitable for multiple treatment choices such as prostatectomy and radiotherapy. No significant difference between surgery and radiation treatment groups regarding the rate of freedom from disease progression and overall survival have been shown in patients with localized prostate cancer [2,3]. Patients living in Japan are eligible for financial assistance from a public medical insurance system for their medical expenses [4]. However, these patients experience a change in their income because of problems such as absenteeism, impaired productivity, early retirement, or compensation at work, which vary depending on their employment situations. Several studies have reported that patients with prostate cancer were less likely to be working within a year after the diagnosis when compared to men without prostate cancer [5,6]. Japan has the oldest population in the world, with 28.1% of the population aged over 65 years in 2018, while the employment rate of males aged ≥ 65 years was on the rise at 34.1% in 2019, which was one of the highest among major countries [7,8]. Employment-related issues are becoming increasingly important among people with prostate cancer. A previous Japanese study reported that the median time from the initial day of sickness absence to a full return to work for male patients with genital cancer was about four months [9]. There is limited information available on the influence of cancer treatments on patients’ employment status in Japan. We hypothesized that the effect of each therapy on patients’ annual income is associated with their type of employment: self-employed, non-regularly employed, and wage and salary workers. Therefore, we conducted an Internet-based survey on the effect of radical treatments (radiotherapy and prostatectomy) for localized prostate cancer on the change in patients’ annual income before and after their diagnoses.

## Methods and materials

In this web-based survey, we sent invitation e-mails to possible participants aged between 20–99 years who were registered as patients with prostate cancer in a medical market research company database, “Macromill Carenet.” Patients were registered under the following contract: they are contacted when the survey starts, their answers are collected anonymously, their answers indicate their consent to the survey, they obtain cashable rewards around JPY100 ($1), and each consent cannot be withdrawn after the survey is completed. Respondents who had undergone radiotherapy or surgery for their localized prostate cancer as the initial radical therapy were included. Participants with any metastasis to the lymph nodes or other organs at their diagnoses, or a history of other cancers were excluded. The survey included questions about the demographic and clinical characteristics at diagnosis and the change in annual income before and after the diagnosis.

Approval for the study protocol was obtained from the Institutional Review Board of the Graduate School of Medicine and Faculty of Medicine, The University of Tokyo (2019363NI).

Categorical variables were compared using the chi-square test. We examined the association of initial radical therapies for prostate cancer, i.e., radiotherapy or surgery, with the change in their annual income before and after their therapies. We defined any increase and decrease within JPY500,000 ($5,000) of annual income as “increase or no change” and decrease of more than JPY500,000 ($5,000) as “decrease.” The elapsed time was defined as the amount of time from diagnosis of prostate cancer to survey completion. Factors affecting the financial toxicity, including income decrease, were known to be low income at baseline, younger age, more recent diagnosis, advanced cancer, education, and employment [10,11]. Furthermore, similar to a previous study, we have added information on the potentially confounding roles of marital status, number of children, and the selected therapies to the variables for the multivariable logistic regression analysis [5]. For the multivariable analysis, the elapsed time was dichotomized by ~5-year, which was determined by a median split technique. All analyses were conducted using R (version 4.0.2). A two-sided P-value of less than 0.05 was considered statistically significant.

## Results and discussion

**Fig 1:**
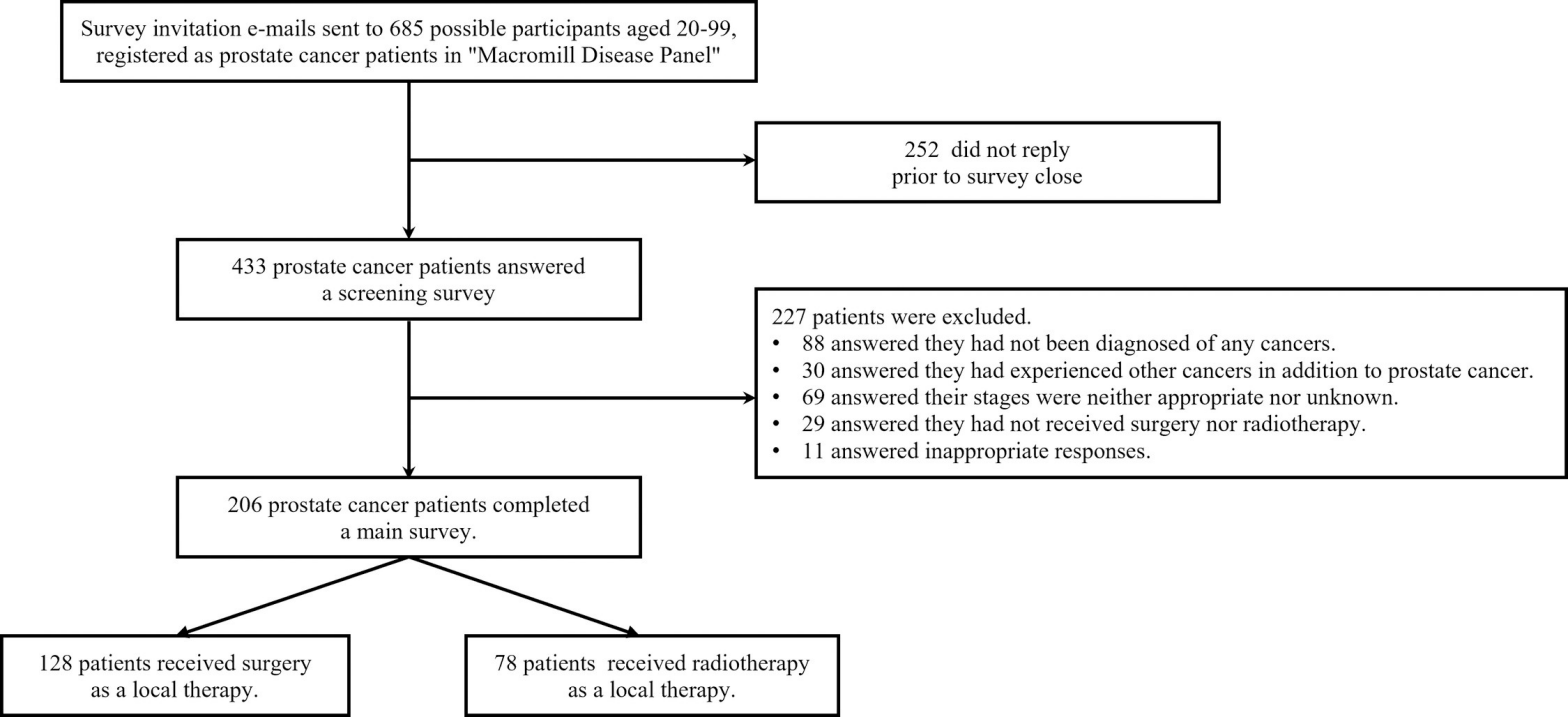
Flowchart of the data collection.

Fig 1 shows the flowchart for data collection. Invitation e-mails were sent to 685 possible participants on April 28, 2020. Among all enrolled patients with localized prostate cancer, we identified 78 who underwent radical radiotherapy (radiotherapy group) and 128 patients who underwent surgery (surgery group); their baseline demographic and clinical factors were not significantly different (Table 1).

**Table 1:** Baseline characteristics between radiotherapy group and surgery group at their prostate cancer diagnosis.

	Treatment				*P* value
	Radiotherapy (N = 78)		Surgery (N = 128)		
	N	%	N	%	
Age group (year)					0.72
<65	35	45%	62	48%	
≥65	43	55%	66	52%	
Marital status					0.60
Single/widowed/div	6	8%	14	11%	
Married	72	92%	114	89%	
Number of children					0.20
Zero	12	15%	11	9%	
Any	66	85%	117	91%	
Education					0.28
High school or less	25	32%	52	41%	
College or more	53	68%	76	59%	
Employment status					0.95
Self-employed worker	10	13%	21	16%	
Non-regularly employed worker	15	19%	13	10%	
Wage & salary worker	25	32%	58	45%	
Jobless	28	36%	36	28%	
Income level					0.60
<JPY4,000,000	16	21%	29	23%	
JPY4,000,000-	31	40%	57	45%	
Unknown/not answer	31	40%	42	33%	
Elapsed time (year)					0.70
<1	9	12%	16	13%	
1 to <3	18	23%	26	20%	
3 to <5	19	24%	31	24%	
5 to <10	24	31%	32	25%	
10 to <20	8	10%	22	17%	
≥20	0	0%	1	1%	
TNM Clinical stage					0.39
cStage I(T1-T2aN0M0)	43	55%	66	52%	
cStage II(T2b-T2cN0M0)	27	35%	40	31%	
cStage III(T3-T4N0M0)	8	10%	22	17%	

Data are presented as the number of subjects in each group with percentages.

The chi-square test showed no significant difference in the “increase or no change” rates of the annual income change before and after their therapies among all employers between the radiotherapy group and the surgery group (78% vs. 71%, P = 0.456). Only among self-employed and non-regular workers, the “increase or no change” rates in the radiotherapy group tended to be higher than that in the surgery group, although with an insignificant difference (88% vs. 58%, P = 0.074).

**Fig 2:**
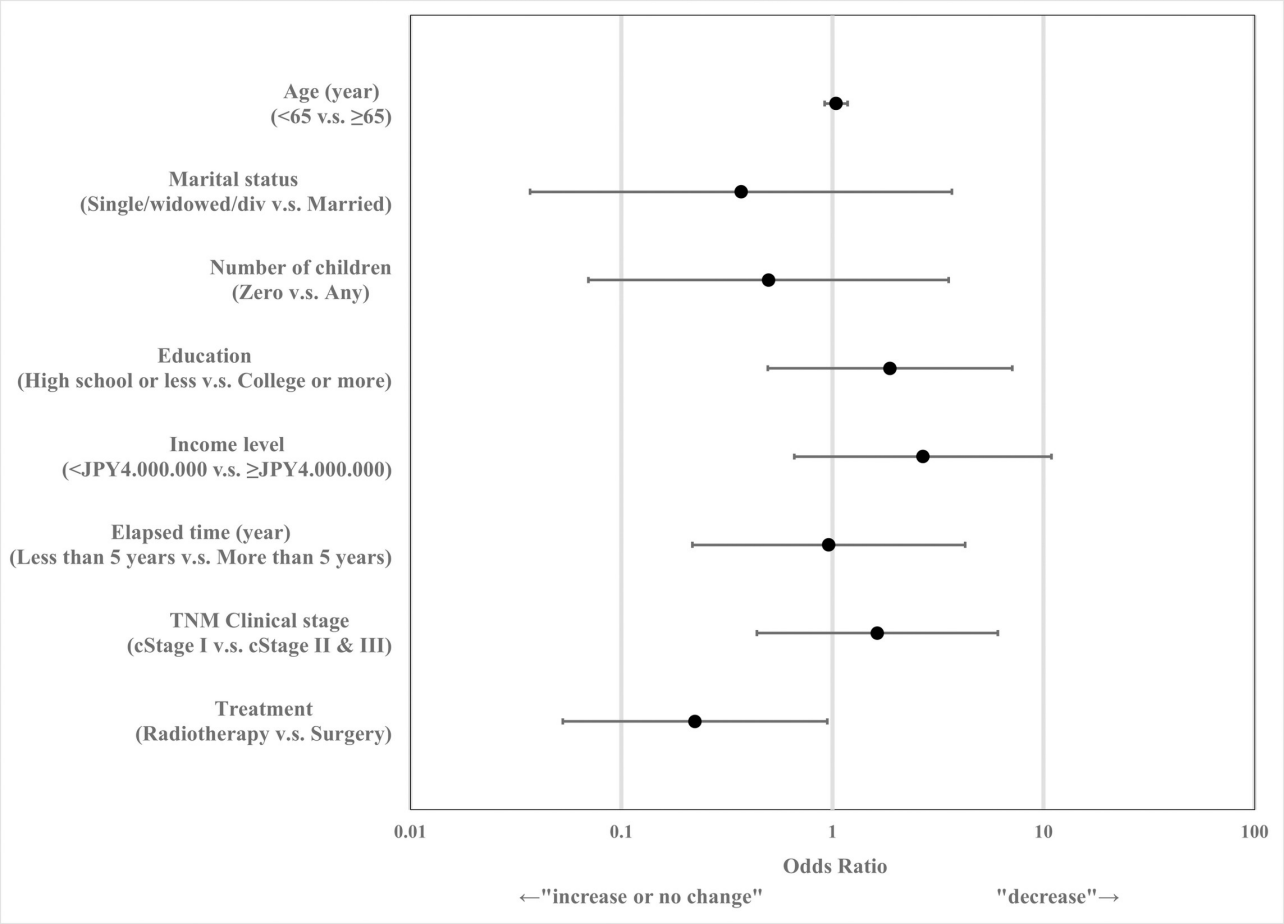
Odds ratio and 95% confidence intervals of annual income change after radical therapy for prostate cancer (JPY4,000,000≈$40,000).

Fig 2 shows the odds ratios (ORs) and 95% confidence intervals (CIs) from logistic regression of the extent of annual income decrease that was adjusted for the characteristics described above. Compared to the surgery group, the radiotherapy group showed approximately a quarter risk of decline in their annual income (OR = 0.22; 95% CI: 0.053–0.946, P = 0.042).

The study investigated post-treatment effects on annual income after radical therapies for localized prostate cancers. The results show that radiotherapy was associated with a smaller risk of decrease in patients’ annual income than surgery among those who had worked as self-employed or non-regularly employed workers.

Previous studies have demonstrated similar results regarding post-treatment effects of radiotherapy on the ability of patients with prostate cancer to return to work [5,12,13]. However, the diversity in global healthcare systems and the universal health coverage prevalent in Japan warranted an evaluation [4]. A Japanese study reported that non-regularly employed survivors were five-fold less likely to return to work, 60% of whom earned a lower income at ≥ 1 year after diagnosis [14]. This study also showed that cancer treatments (surgery, radiotherapy, and chemotherapy) were not associated with patients’ return to work [14]. Interestingly, our study showed that radiotherapy had a positive effect on the finances of patients with prostate cancer with a specific employment status: self-employed or non-regularly employed. Moreover, the comparison of income change among patients with prostate cancer and the general population with similar patient characteristics would also be an important issue in the future.

Our results may be socio-medically plausible from the following explanation. Hospitalization for surgery requires patients to take sick leaves. While wage and salary workers would be compensated by their employers, a lower probability of compensation among self-employed and non-regularly employed workers would lead to lower revenues. Contrarily, radiotherapy in an outpatient setting may help avoid decreases in income for workers with few social guarantees by coordinating treatment schedules. Practically, from a public report in 2013, most Japanese corporations were equipped with sick leave systems, but only 48.5% allowed non-regularly employed workers to use the systems [15]. According to another public survey in 2019, more than 40% of self-employed workers reported feeling a lack of unemployment insurance when they lose their jobs [16]. No reports support our hypothesis, which emphasizes the novelty of our research. In addition, the reason for the decline in the ORs for the higher income-level shifted may be that patients with higher earnings had relatively more savings, thereby being less resistant to take sick leaves, or their higher wages per hour led to immense losses even at shorter absences.

The advantage of avoiding decrease of income after radiotherapy will be more outstanding in Japan because of the increased likelihood of prostate cancer in Japanese workers and a prevalence of stereotactic body radiotherapy (SBRT) to localized prostate cancers in the future [1,8]. In 2016, the Ministry of Health, Labor and Welfare approved use of SBRT as insurance coverage, which provides five or fewer fractions with almost equivalent effectiveness and requires fewer sick leaves [17–19].

Several limitations of this study should be acknowledged. The response rate was relatively low, as shown in Fig 1. The small number of cases was one of the most important limitations of the present study. Due to the small sample size, there was a high risk of confounding variables in our statistical model. More cases are needed before our results can be extrapolated to a larger population. We could not include detailed clinical information on the treatments and laboratory data such as radiation dose, androgen deprivation therapy, the Gleason score, and serum prostate-specific antigen level because our survey was conducted by a third-party company, which meant that the information they gathered was completely separate from the medical health records of the hospital. To address the limitations of the small sample size and the insufficient clinical data in this study, we should recruit and investigate as many patients as possible in clinical settings. Questions about their quality of life and adverse events associated with the treatments were not included. The clinical information, such as diagnosis and clinical stages, might be inaccurate because the present study was based on an anonymized patient-based survey. Unmeasured confounders could have possibly affected our multivariable logistic regression models. Despite these limitations, we believe that our results are socio-clinically important. Currently, we positioned this study as a pilot one and are planning to carry out a large-scale study in the clinical settings of our hospital based on the results of this study. Further research about the dynamics of income change after the treatment of prostate cancer is also necessary in the future.

## Conclusions

We performed a multivariable logistic regression analysis using a web-based survey of patients with localized prostate cancer and compared the post-treatment decline in income after radical radiotherapy and surgery depending on employment status. Radiotherapy showed less than a quarter risk of annual income decreases after treatment. Further studies are required to test our findings in clinical practice in the future.

## Supporting information

S1 Dataset (XLSX)
